# Beyond the high mortality burden: targeting quality of life in Brazil

**DOI:** 10.1590/S1516-31802002000300001

**Published:** 2002-05-02

**Authors:** Isabela M. Benseñor, Paulo A. Lotufo

Until forty years ago, few physicians were aware of the relevance of studies addressing quality of life. However, by 2001, more than 40,000 papers about quality of life had been published. It is now clear, for many physicians and scientists around the world, that it is not enough for people just to be alive, but that they should be able to live with pleasure and comfort.

It is not easy to define quality of life, and the methodology for studies regarding this have no clear concepts and operational definitions that can be used as standards. Marcia Testa, in a seminal review of the methodology used in quality of life studies, published in the New England Journal of Medicine,^1^ very ably discussed all the pitfalls in studies addressing quality of life. The most important of these is that quality of life is not directly measurable in the way that blood glucose level is. Quality of life is measured indirectly using questionnaires such as the SF-36 (Short Form #36), the WHO-QOL World Health Organization Quality of Life instrument, SAS,^2-4^ and others. These apply elementary questions about daily activities, limitations to physical activity, changes in family life, and alterations in mental health. By simply asking questions, they try to infer the patient's quality of life in relation to some disease or in their usual life. There are also some questionnaires designed to measure quality of life for specific diseases, such as the St George Hospital questionnaire, which is commonly used for patients with chronic obstructive respiratory disease. Sometimes, investigators can use a general questionnaire together with a specific questionnaire for some disease, in an attempt to be more sensitive and specific. But we do not know whether such questionnaires are really measuring what they say they are.

We now have a lot of papers about quality of life in chronic diseases like chronic obstructive respiratory disease, chronic heart failure, or various types of cancer in developed countries. Some of these questionnaires are available in Brazil, translated and validated with very good methodology. What would be our position regarding quality of life studies and quality of life data, here in Brazil?

The official health data show that Brazilians, just like most other people in the world, have cardiovascular disease as their main cause of death. In the Northeast and North of our country, stroke is the most important cause of death among the cardiovascular diseases. In the more affluent states of Brazil, the mortality caused by coronary heart disease is higher than for stroke, although the burden of mortality is premature in comparison with developed countries. Thus, if we have no control over quantity of life (or quantity of death), is it relevant to worry about quality of life? Yes, it is, because once again, staying alive is not the only goal we have to reach in Public Health. It is fundamental, not only to be alive, but to live well. People are not zombies in a terror movie. People need to live without pain or discomfort, and with peace and dignity.

So, it is very important to study quality of life in our country, and this can be a new goal for medical care and also for research in this new millennium. Quantity of life and quality of life have to go together and cannot be divided, in the same way that we, all health professionals, cannot divide body and soul into different things within the same person. Again, merely being alive is not enough: all people need to live well.

## References

[1] Testa MA, Simonson DC (1996). Assessment of quality-of-life outcomes. N Engl J Med.

[2] Gandek B, Ware JE, Aaronson NK (1998). Tests of data quality, scaling assumptions, and reliability of the SF-36 in eleven countries: results from the IQOLA Project. International Quality of Life Assessment. J Clin Epidemiol.

[3] Bonomi AE, Patrick DL, Bushnell DM, Martin M (2000). Validation of the United States’ version of the World Health Organization Quality of Life (WHO-QOL) instrument. J Clin Epidemiol.

[4] Weissman M, Bothwell S (1976). Assessment of Social Adjustment by Patient Self-report. Archives of General Psychiatry.

